# Correlation between delayed-enhancement magnetic resonance and nitrate myocardial Tc-99m tetrofosmin scintigraphy in myocardial infarction: a case report

**DOI:** 10.1186/1752-1947-1-120

**Published:** 2007-10-30

**Authors:** Mauro Feola, Gian Luca Rosso, Alberto Biggi, Stephane Chauvie, Giovanni Leonardi, Franca Margaria, Maurizio Grosso, Valeria Ferrero

**Affiliations:** 1 Department of Cardiovascular Diseases, Ospedale Santa Croce-Carle Cuneo, Italy; 2 Nuclear Medicine Service, Ospedale Santa Croce-Carle Cuneo, Italy; 3 Radiology Service, Ospedale Santa Croce-Carle Cuneo, Italy; 4 Division of Cardiology Universita' di Verona Ospedale Borgo Trento Verona, Italy

## Abstract

**Introduction:**

Delayed-enhancement magnetic resonance imaging (DE-MRI) has been recently proposed as an alternative tool in identifying myocardial viability and transmural distribution of necrosis in the myocardium.

**Case presentation:**

We describe a case of a 71-year-old man admitted for ischemic-like chest pain in which DE-MRI and post-nitrate ^99m^Tc-tetrofosmin myocardial scintigraphy equally contributed to the diagnosis of previous lateral myocardial infarction.

**Conclusion:**

In this patient with coronary artery disease, the absence of uptake of tracer at myocardial scintigraphy appeared to be closely correlated to DE-MRI data. Cardiologists can use SPECT or DE-MRI to obtain similar information about myocardial viability.

## Introduction

Delayed-enhancement magnetic resonance imaging (DE-MRI) is a highly accurate method for the non-invasive estimation of infarct size and location in acute or chronic myocardial infarction [1-3]. Cardiac MRI can also be used to detect the chronic consequence of myocardial infarction using its high degree of image definition [4]. It has recently been proposed as an alternative tool for the assessment of myocardial necrosis. Diagnostic methods for assessing myocardial viability, such as positron-emission tomography, single-photon-emission computed tomography (SPECT) and dobutamine echocardiography all have demonstrated limits in identifying the transmural extension of necrosis and/or viability of the ventricular wall [5-7].

Previous studies [1,8,9] have documented the comparable efficacy of myocardial SPECT (using either thallium 201 or ^99m^Tc-tetrofosmin) and DE-MRI in the evaluation of the presence, location, and transmural extension of myocardial necrosis. Furthermore, DE-MRI is a diagnostic method that is characterized by superior spatial resolution and the absence of ionizing radiation.

In this case report we describe the case of a man with a previous lateral myocardial infarction, in which MRI showed a DE in the lateral wall according to the result of post-nitrate ^99m^Tc-tetrofosmin imaging.

## Case presentation

**Figure 1:**
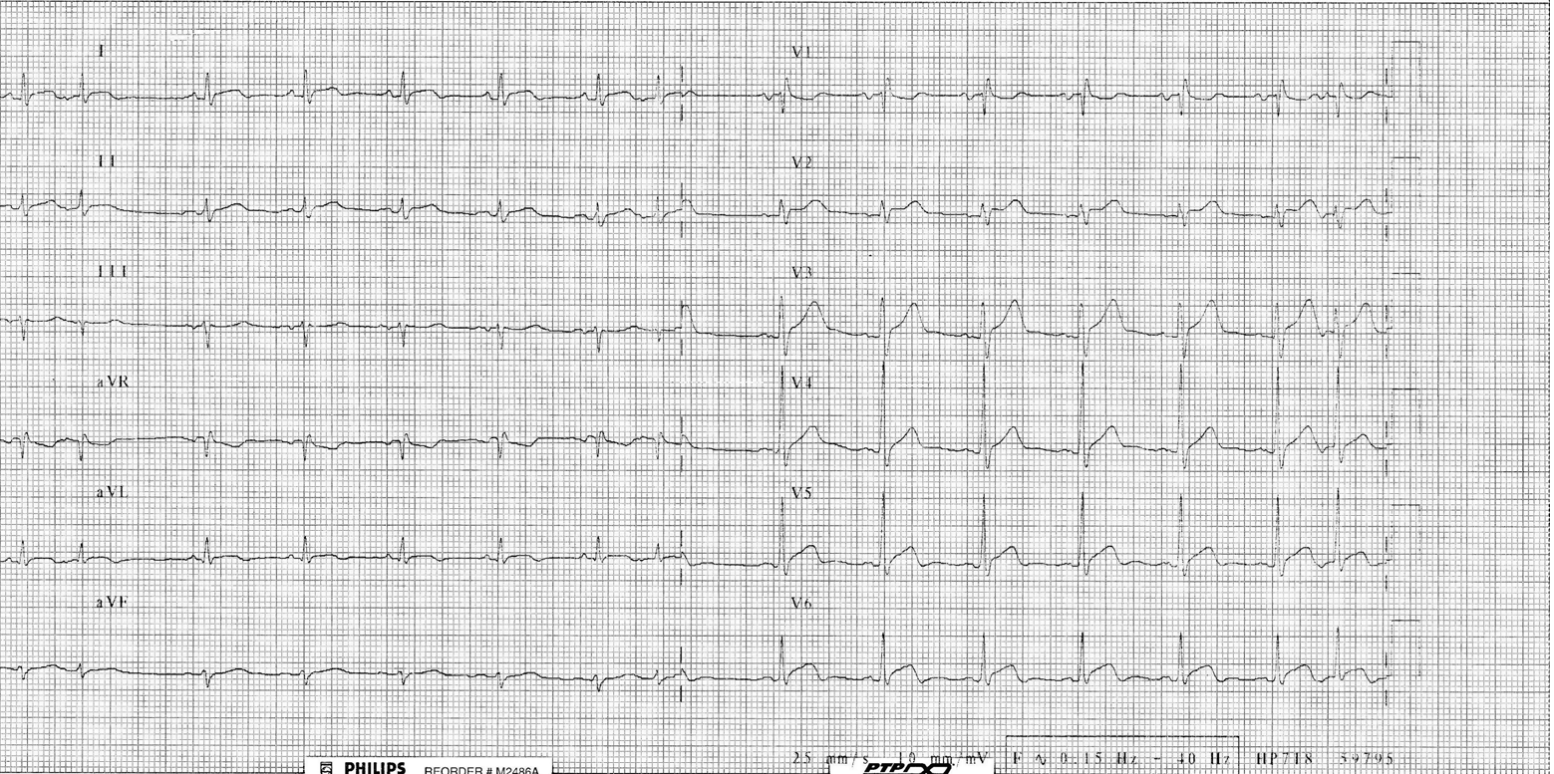
The 12-lead electrocardiogram showed persistent ST-T segment elevation in the infero-lateral leads, supraventricular premature complexes and incomplete right bundle branch block. This ECG may identify either a non-q myocardial infarction or pericarditis.

**Figure 2:**
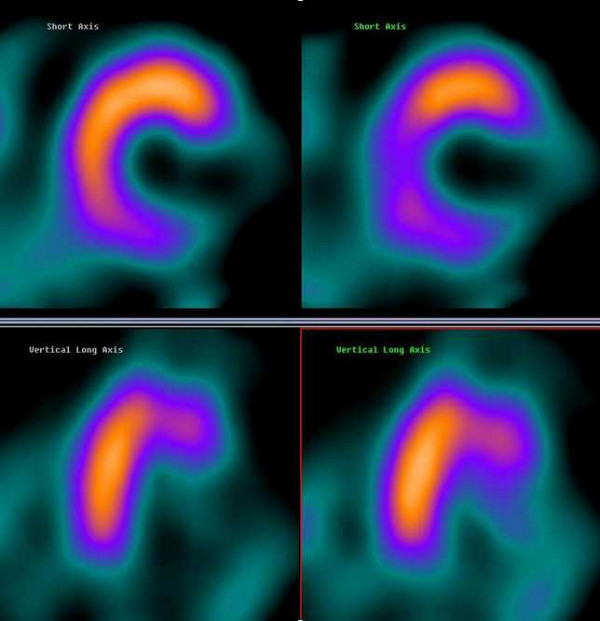
Post-nitrate myocardial (left images) and rest (right images) ^99m^Tc-tetrofosmin SPECT showing absent uptake of tracer in lateral and infero-lateral wall. The nitrate administration did not significantly change the perfusion pattern.

**Figure 3:**
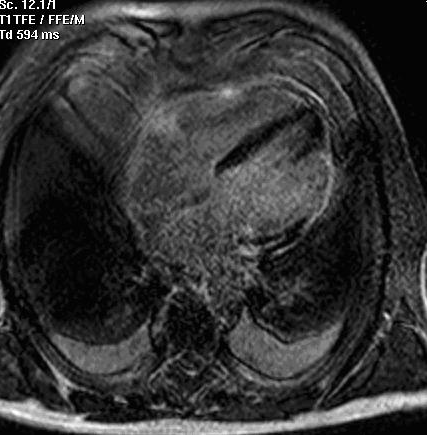
Cardiac MRI after gadolinium injection demonstrated a transmural area of DE in the lateral wall of the left ventricle. A bilateral pleural effusion was also evident.

A 71-year-old man was admitted for ischemic-like chest pain occurring 10 days prior to the hospital admission. The ECG showed ST-T segment elevation without a q wave in the infero-lateral leads (figure 1) that could identify either myocardial infarction changes or pericarditis. A persistent release of cardiac markers was registered (troponin I 1.05 μg/l [normal value< 0.6 μg/l]) together with a slight increase in inflammatory markers and white blood cells. The clinical presentation, the ECG pattern and the laboratory data were not sufficient to rule out a diagnosis of pericarditis. Transthoracic echocardiography was not diagnostic. The patient underwent two ^99m^Tc-tetrofosmin myocardial SPECTs on different days: the first at rest and the second after the use of nitroglycerine (0.005 mg/kg per os). The left ventricular ejection fraction (LVEF) was calculated using a previously validated and commercially available automated software (quantitative gated SPECT, QGS, Cedars-Sinai Medical Center, Los Angeles, CA [10]). The oral post-nitrate images clearly demonstrated an absence of uptake of ^99m^Tc-tetrofosmin in the lateral and infero-lateral wall (figure 2); the LVEF was 31%. This absence of tracer uptake after the nitrate administration clearly indicated the presence of non-viable myocardium [11]. The same patient underwent cardiac MRI using a clinical 1.5-T Gyroscan ACS-NT MRI scanner (Philips Medical System, Eindhoven, The Netherlands). The scan was analysed according to: a) the left ventricular function (sequences balanced-echo cine MRI) and b) the presence of scar tissue with delayed-enhancement (DE) images. Delayed sequences were obtained approximately 12 minutes after intravenous injection of 0.2 mmol/kg gadolinium diethyltriaminepentaacetic acid (Gd-DTPA) using a fast field echo sequence (slice thickness 8 mm, FOV 360 mm, flip angle 15°, TE 1.3 ms, TR 4.1 ms). Delayed enhancement images were displayed with a grey scale to optimally show normal myocardium (dark) and the region of delayed-enhancement myocardium (bright). The akinesia of the infero-apical left ventricular segment and the diskinesia of the lateral wall emerged (figure 3). The LVEF was calculated as 36%; a bilateral pleural effusion was also present. The DE distribution was transmural with a dilatation of the lateral wall that appeared like an aneurysm. Necrotic myocardium usually accumulates and retains gadolinium-based contrast material for 10 or more minutes after the agent has been administered [12]. Considering the results of myocardial SPECT and MRI together, coronary angiography was performed on the fourth day and occlusion of left circumflex coronary artery was identified. As a result of the clinical and image data, medical therapy was continued.

## Discussion

The DE MRI technique has quickly been found to have potential utility as an important clinical method of evaluating cardiac viability in ischemic myocardium [3,12]. In this case report the severity of the perfusion defect identified at myocardial SPECT after nitrate assumption closely correlated to the transmural distribution of DE-MRI. In 21 patients with severe ischemic left ventricular dysfunction, Giorgetti et al [8] demonstrated a clear correlation between the extension of ^99m^Tc-tetrofosmin myocardial SPECT uptake injected during nitrate infusion and the distribution of necrosis at DE-MRI. The infusion of nitrate significantly increased the tracer uptake at SPECT scan in DE-MRI viable segments, improving the diagnostic accuracy in the detection of myocardial viability. In our patient we obtained the same information after sublingual nitrate administration before the tracer injection: this convenient method of drug administration is faster and might help in a clinical setting. Evidence of persistent viability after myocardial infarction not treated with reperfusion procedures should always be investigated. In fact, the OAT study did not demonstrated any clinical utility of percutaneous revascularization in stable patients with persistent total occlusion of the infarct-related artery [13].

## Conclusion

In conclusion, the oral postnitrate ^99m^Tc-tetrofosmin myocardial SPECT has been confirmed to be closely correlated with DE-MRI images indicating their comparable value to estimate myocardial viability. In patients with coronary artery disease, clinicians might obtain similar information about myocardial viability using either myocardial SPECT or DE-MRI.

## Consent

We wish to acknowledge that the patient provided written consent for publication of this case report and these images.

## Competing interests

The author(s) declare that they have no competing interests.

## Authors' contributions

MF and GLR collected data and drafted the manuscript. AB and SC analysed the SPECT scan. GL and FM performed the cardiac MRI. MG and VF proofed the final version of the manuscript. All authors read and approved the final manuscript.
